# Avoidant/Restrictive Food Intake Disorder in a Pediatric Obese Patient

**DOI:** 10.7759/cureus.76390

**Published:** 2024-12-25

**Authors:** Paolo A Zegarra-Lizana, Elizabeth J Ramos- Orosco, Daniel Cisneros, Efrain Flores

**Affiliations:** 1 School of Medicine, Universidad Peruana de Ciencias Aplicadas, Lima, PER; 2 Pediatrics, Nationwide Children’s Hospital, Columbus, USA; 3 Principles and Practice of Clinical Research, Executive and Continuing Education (ECPE) Harvard School of Public Health, Boston, USA; 4 Pediatrics, Dr. Efrain Flores Pediatrics, Bolingbrook, USA

**Keywords:** arfid, avoidant-restrictive food intake disorder, “childhood obesity morbidity”, obesity counseling, obesity practice

## Abstract

Avoidant/restrictive food intake disorder (ARFID) can present with limited food variety, intake, or aversions. The symptoms can manifest at any age and typically appear in the first few years of life. The prevalence of ARFID varies widely among clinical and non-clinical populations, and its diagnosis requires trained health professionals to ensure early detection and prevention of poor outcomes.

A four-year-old boy developed an aversion to solid foods after a choking incident with chicken nuggets, fearing a recurrence. Pre-existing phobias and developmental delays compounded his selective eating. Despite a BMI percentile over the 99^th^ percentile for his age (20.34 kg/m^2^), ARFID was diagnosed after a psychiatric referral, highlighting the intricate psychological aspect of pediatric feeding disorders.

Patients with ARFID may exhibit unexpected weight variations, and nutritional deficiencies do not always appear with low body weight. Child obesity is a pressing US public health issue, affecting 19.7%, potentially leading to psychiatric comorbidities such as depression or anxiety. Health professionals require training to detect and prevent adverse outcomes.

Understanding the mechanisms perpetuating ARFID and addressing mental health in this population are crucial aspects of daily practice.

## Introduction

Since 2013, avoidant/restrictive food intake disorder (ARFID) has been identified as a psychiatric condition classified in the DSM-5. This disorder presents with abnormal eating behavior with persistent failure to meet appropriate energy or nutritional needs, not explained by other medical or non-medical conditions [1]. These odd eating habits may arise from an inability to tolerate specific sensory properties of food, such as texture or taste; unreasonable fear of the consequences of eating, such as vomiting or choking; and a general disinterest in food or eating [2].

The prevalence of ARFID is estimated to range from 5% to 14% [3], with a higher number of affected children between the ages of four and nine years, particularly males, and with a longer duration of illness compared to other eating disorders [4]. However, with heterogeneous data about the setting and sample characteristics, there is literature with values that go from below 1% in general population samples from European and Asian countries to up to 64% in a tertiary care pediatric feeding clinic and an estimated incidence from a national surveillance study of 2.02 per 1,00,000 children and adolescents in Canada. Common psychiatric comorbidities observed in children diagnosed with ARFID include anxiety disorders and autism spectrum disorder. Nonetheless, this group of patients seemed to be less prone to mood disorders than other eating disorders [5].

Based on nutritional deficiencies, patients may present with clinical signs such as fatigue, syncope, constipation, hair loss, abdominal pain, hypothermia, bradycardia, and paleness, among others [2]. Additionally, an impact on growth and bone development has been observed, reflected in reduced longitudinal growth, which is suspected to be related to deficiencies in proteins, zinc, calcium, vitamin D, and dairy intake [6].

The diagnosis of ARFID still needs to be completely clear, as there is no standardized approach, and it can be confused with other psychiatric disorders. Various assessments are used, such as the Eating Disturbances in Youth Questionnaire (EDY-Q) [7], DSM-5 criteria, and Stanford Feeding Questionnaire [8], among others. However, standardization is yet to be achieved. The symptoms associated with the diagnosis can be grouped to prepare for its management in three groups: limited variety, limited ingestion, and aversive [9]. 

Here, we present a case of a four-year-old boy diagnosed with ARFID. We highlight some mechanisms involved in this condition, the importance of physical and mental health in this population, and the awareness of a not-so-common presentation of malnutrition that is increasing in frequency.

## Case presentation

This clinical case involves a four-year-old boy who presented with an onset of four months of poor appetite for solid foods following a choking incident with chicken nuggets. The episode, which required a Heimlich maneuver by the mother, resulted in the child's reluctance to consume solids and a preference for liquids only during the following two weeks, prompting the parents to resort to a diet of liquefied foods. This group included commercial nutritional supplements such as four bottles of milk per day that the family reduced to eight ounces daily, shakes, jello, and blended bananas. The patient exhibited fear of choking, compounded by pre-existing phobias related to hair cutting and insects. This aversion to solid foods raised concerns about potential nutritional deficiencies, so the parents scheduled a visit to the pediatrician.

About his past medical history, he was born by spontaneous vaginal delivery without any complications during pregnancy. The child also had a medical history of developmental and speech delay, as well as aggressive behavior, for which therapeutic interventions around two years old included physical therapy, occupational therapy, and speech therapy. In addition, he had a neuropsychological evaluation done at three years old. However, they could not confirm autism spectrum disorder (ASD) nor attention deficit hyperactivity disorder (ADHD), and they recommended he return at age five.

Physical examination yielded unremarkable vital signs with a BMI of 20.34 kg/m² (above the 99^th^ percentile), while an X-ray of the neck revealed a clear airway with no foreign bodies. In addition, his laboratory results showed in-range folate and vitamin B12 levels (23.1 ng/ml and 1820 pg/ml, respectively) and slightly decreased vitamin D levels (25 ng/ml). Normal values: 30 to 100 ng/ml.

A referral to psychiatry was made, leading to the diagnosis of avoidant/restrictive food intake disorder. The specialist scheduled an appointment with the neuropsychologist and clinical psychologist and a follow-up with his primary care pediatrician in six weeks. During a follow-up visit at around five years old, the patient started psychotherapy at Lurie's Hospital, and at that time, the mother had to schedule a neuropsychological evaluation. He started eating better and received support from school and his speech therapist.

**Figure 1:**
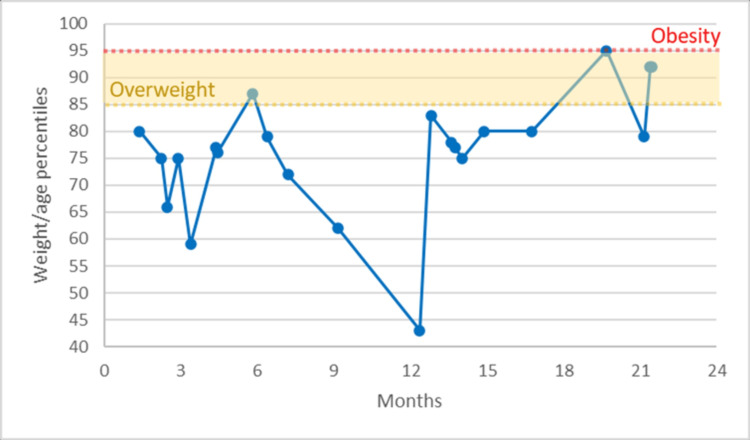
WHO weight-for-age percentiles in the first two years WHO: World Health Organization

The graphs present the changes in weight over time. In Figure 1, the weight per age percentile measures showed a peak of overweight around six months old that started falling to typical values until the first year. After that, the patient's weight increased again, reaching values near the upper percentiles. In the last months of his second year, those values oscillated between overweight and obesity. 

**Figure 2:**
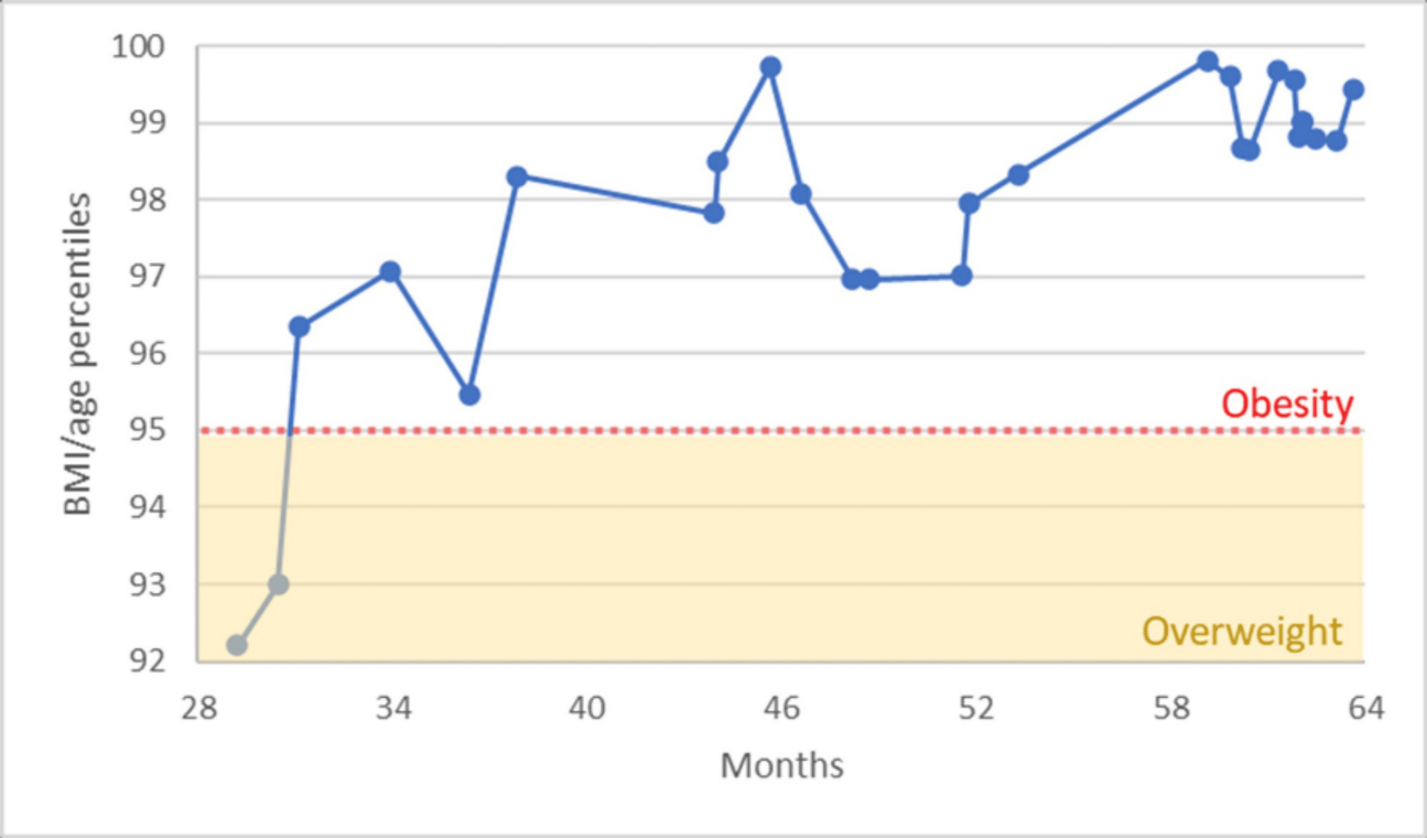
CDC extended BMI for age percentiles (2 to 6years) CDC: Centers for Disease Control and Prevention, BMI: Body Mass Index.

After two years of age, the BMI per age percentiles of the patient showed in the graph of Figure 2 that the patient continued the trend from overweight to finally reaching the obesity category at 31 months of age. However, there are some ups and downs in this range of percentiles. The highest peak occurred when the patient was 46 months old, and after that, followed an important down to the 97th percentile. After the first visit with his pediatrician at 53 months, the trend started to repeat until another peak at around 60 months. At that time, the patient visited psychiatry, and the values of BMI/age stopped growing and did not surpass the previous marks until the last visit reported at 63 months.

## Discussion

In this case, the patient presentation is similar, as expected, to the literature reviewed with a four-year-old boy with a history of phobias and aggressive behavior. As reviewed by Sanchez-Cerezo et al., this condition is more common in young males with usual psychiatric comorbidities such as ADHD, ASD, or anxiety disorders [5,10,11]. The only confirmed condition in this patient was his specific phobias to hair cutting and insects. However, health professionals should be aware of some aspects of the disease.

ARFID is a disorder that, since its definition, has helped to elucidate different strategies to manage some presentations related to food ingestion in children. The aversive presentation for the diagnosis usually comes after a traumatic event that is afraid of being repeated, not necessarily associated with feeding. Alongside some anxiety-related features and hypersensitivity to corporal sensations, the initial fear led to overestimating the probability that this event would repeat in time or with different kinds of food. In severe cases, all the solids can be limited, and only soft or liquid food can be consumed. Another interesting feature of this group is that it can present during any moment of life, compared with the groups of limited variety and limited ingestion of ARFID, which are more likely to appear during the first years [9,12,13]. In this case, the patient had more symptoms of a severe clinical presentation of aversive ARFID without tolerance for solid foods.

The expected body mass of patients with ARFID is typically underweight, although milder cases can be diagnosed without such a pronounced change. Yule S et al. studied nutritional deficiencies in ARFID associated with ASD, observing that these deficiencies did not always correlate with low body weight. 14.3% of 35 patients were overweight or obese, with normal-range weight values in most of the rest [14]. This case report places the patient in the obesity category based on their BMI for age with persistence since he was two years old (Figure 1), reflecting a growing trend in the country.

Child obesity is one of the biggest concerns in public health in the United States. The prevalence between the ages of two and 19 is approximately 19.7%, representing nearly 14.7 million children and adolescents. Despite a lower prevalence in the early ages of 2-5 years (12.7%), as a chronic condition without management, the consequences in the world would add up [15]. In 2022, Ling J et al. studied the worldwide economic burden of overweight and obesity in children compared to those with normal weight. They found an increased annual total medical cost of up to $237.55 per capita attributable to high weight, with the annual direct and indirect costs projected to be $13.62 billion and $49.02 billion by 2050 [16]. One of those indirect costs may lean upon psychiatric comorbidities, which are higher in this group of patients, as pointed out by Wen-Wang Rao et al. They found that obese children and adolescents have more risk for major depressive disorder in comparison to healthy controls, like the increased prevalence of depressive and anxiety symptoms described by Wang S et al. in China [17,18]. Therefore, mental health in this population should also be an object of screening in daily practice.

Also, patients with malnutrition frequently have micronutrient deficiency, which limits children from reaching their full potential in cognitive and intellectual abilities, especially with nonverbal reasoning and logical problem resolution [19]. Therefore, Tam E et al. described in their study that some diseases directly involve micronutrients. Multiple interventions with micronutrients can reduce anemia, lipid-based nutrient supplementation (LNS) can reverse stunting and underweight, vitamin A can reduce general mortality, and zinc decreases diarrhea incidence [20]. This mineral supplementation in premature babies also helped to reduce mortality and stimulate early growth [21].

In mental health, Głąbska D et al. describe vitamin D as a major player in several conditions; therefore, they recommend maintaining average blood values to prevent or alleviate those diseases [22]. This patient presented low in vitamin D; as the previous study suggests; either the vitamin D deficiency or his aggressive behavior and suspicion of ASD could have influenced the other. Adequate supplementation might be beneficial in this case, and physicians should keep it in mind during the holistic evaluation of patients with ARFID.

Additionally, the mechanism to perpetuate its condition seemed similar to people without the ARFID diagnosis, as studied by Kerem et al. The reward surfeit theory of overeating postulates that an increase in the reward response of regions in the brain can lead to excess consumption of high-calorie food without a homeostatic need. Furthermore, human functional magnetic resonance imaging (fMRI) studies in people who were overweight/obese showed hyperactivation of a neurologic circuit of food motivation in response to some appetizing food. These areas include the insula, orbitofrontal cortex (OFC), and nucleus accumbens. Kerem et al. found that in patients with ARFID and obesity, the OFC area was more activated in response to subjective hunger than in ARFID patients with normal weight [23]. Therefore, the ingestion behavior of ARFID among these patients can potentially increase the metabolic consequences of adiposity in the patient reported, which predisposes them to maintain obesity and include him in a more vulnerable population.

There is insufficient scientific evidence to establish the definitive management of this syndrome, but most publications agree on a multidisciplinary approach. Bialek-Dratwa A. et al. describe methods such as food chaining, family-based treatment, cognitive-behavioral therapy (CBT), or feeling and body investigators [24]. Their review focuses more on the behavioral and nutritional approaches, where understanding the patient is key for the therapeutic process. Del Toro V et al. also highlights the role of the mental health specialist for most cases, suggesting the same multidisciplinary approach for the more severe cases denoted by red flags and signs of impaired oral development [9]. Both studies did not suggest pharmacological management for this condition and agreed that further clinical studies should be done to clarify more of the therapy. 

Therefore, psychotherapy plays an essential role in the management of ARFID, with a particular incidence of CBT among the many alternatives. Wilmott E et al. reviewed all the currently used therapies for this diagnosis. These included behavioral approaches, CBT, family-based therapy, and a combination of these across life spans and clinical environments. As with other management decisions, the best one for each patient should consider its demography, trigger and maintaining factors of ARFID, comorbidities, etcetera [25]. For example, in those under age 21, the family therapy interventions were more applied. In this patient, the behavioral approach in his group is one of the most used for those under age 15, especially with escape extinction techniques in younger children. This is part of the CBT approach and improves sensorial sensibility, the fear of adverse consequences, and all the other benefits of losing interest/appetite from the other therapy components [26]. Therefore, it would be an excellent option for the patient to follow, as it would target the particular type of ARFID in this case. However, we should hope for the best for the patient's care among his available options to continue the treatment for the appropriate age group.

In this case, the management included a multidisciplinary approach with the participation of the pediatrician, neuropsychologist, clinical psychologist, occupational therapist, and speech therapist, without using any psychotropic drugs for the patient. Figure 2 shows that it stopped the upward trend of weight gain from previous years. Based on the gathered literature, this approach is according to the current general management for this diagnosis.

## Conclusions

This case highlights a complex and atypical presentation of ARFID in a four-year-old boy with severe aversive eating behaviors, specific phobias, and obesity. Although ARFID is often associated with underweight or malnourished patients, this patient demonstrates how selective food avoidance can contribute to weight gain, emphasizing the importance of recognizing diverse clinical presentations. The successful stabilization of the patient’s weight trajectory reinforces the value of a multidisciplinary, non-pharmacological approach.

The intersection of ARFID, psychiatric comorbidities, and atypical nutritional patterns underscores the need for early identification and intervention. Understanding the mechanisms perpetuating ARFID and addressing mental health in this population is crucial for improving the quality of life of these patients. Therefore, we encourage further research to detect this condition early, even in atypical presentations.
